# Revolutionizing cancer immunotherapy: T cell engagers beyond hematologic malignancies toward solid tumors

**DOI:** 10.3389/fimmu.2026.1774471

**Published:** 2026-05-20

**Authors:** Xinyi Wu, Ping Li, Tao Li, Qiwen Fan, Hao Wu

**Affiliations:** 1 Department of Oncology, The First Affiliated Hospital of Nanjing Medical University, Nanjing, China; 2 Department of Gastric Cancer Center, The First Affiliated Hospital of Nanjing Medical University, Nanjing, China; 3 First Clinical Medical College, Xuzhou Medical University, Xuzhou, China

**Keywords:** combination therapy, solid tumors, T cell engager, trispecific antibody, tumor microenvironment

## Abstract

T cell engagers (TCEs) are a class of T cell–redirecting therapeutics that enhance antitumor immunity by bringing T cells into close proximity with malignant cells. Following their success in hematologic malignancies, TCEs are now being increasingly investigated for the treatment of solid tumors. The recent approval of tarlatamab for small cell lung cancer (SCLC) offers renewed hope in this setting. Nevertheless, clinical efficacy in solid tumors remains limited by immunosuppressive tumor microenvironments (TME), on-target/off-tumor toxicity, and intrinsic or acquired resistance to TCEs. This review summarizes recent advances in TCE development for solid tumors, including refinements in molecular design, biomarker-guided patient selection, and rational combination strategies aimed at overcoming resistance and improving therapeutic outcomes. We also discuss emerging next-generation approaches, such as engager platforms that redirect other immune effector cells. Collectively, these innovations underscore the potential of more precise and effective engager-based therapies for solid tumors.

## 1 Introduction

Immunotherapies revolutionized cancer treatment by restoring effective immune function to kill tumors and demonstrated significant efficacy across various cancer types. However, traditional immunotherapies had limitations, including low immune infiltration, poor response rates, and limited T cell activity (1).

**Figure 1:**
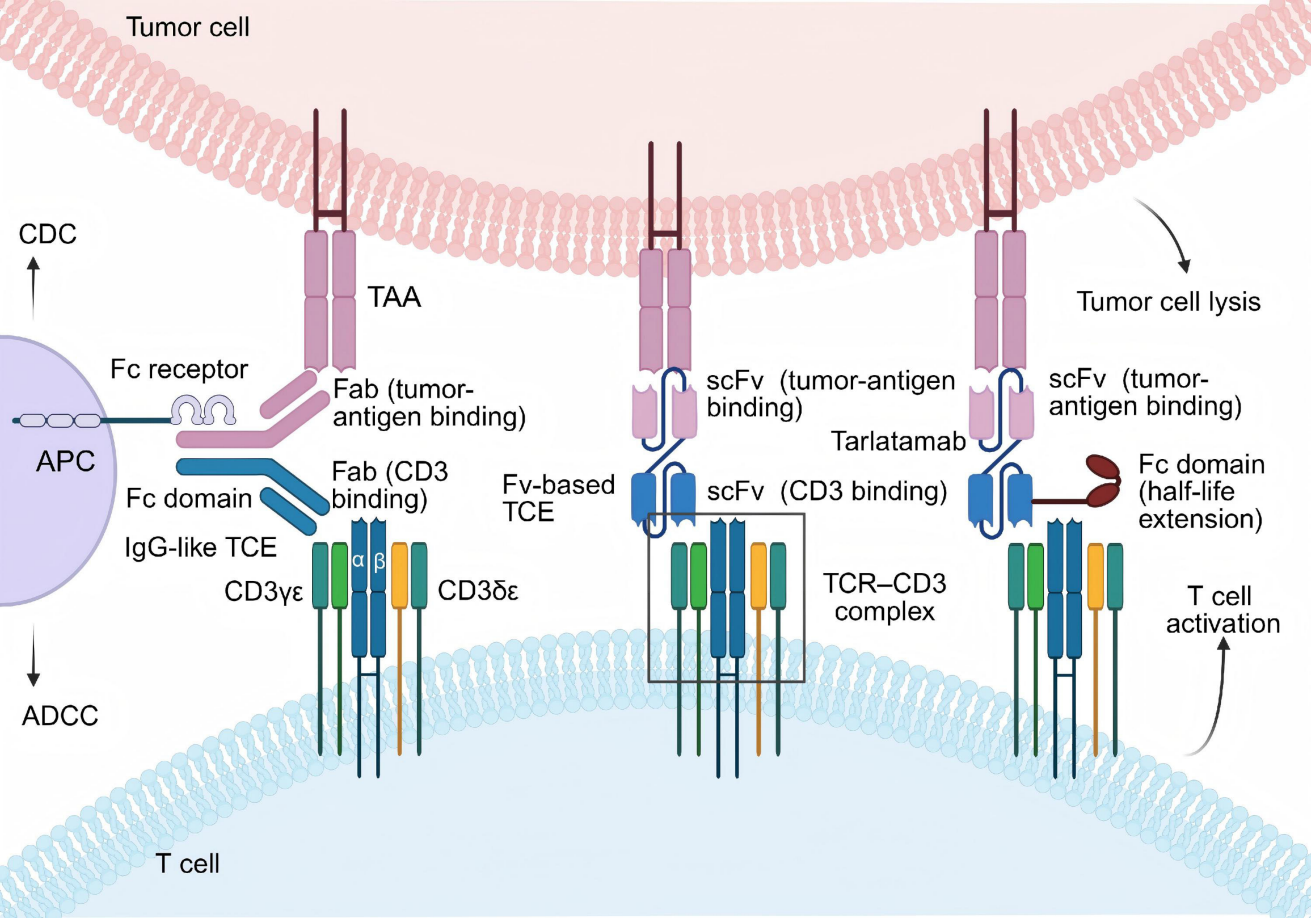
Representative formats and mechanisms of T-cell engagers (TCEs). TCEs bridge a tumor-associated antigen and CD3 on T cells to form an immune synapse, activate T cells, and induce target cell lysis. Left: IgG-like TCEs retain an Fc region; one Fab targets the tumor antigen and the other targets CD3; Fc interactions with Fc receptors on antigen-presenting cells can modulate pharmacokinetics and enable effector functions (ADCC/CDC). Middle: Fv-based TCEs are tandem scFv without an Fc, providing minimal spacing at the immune synapse. Right: Tarlatamab is a bispecific TCE that binds delta-like ligand 3 on tumor cells and CD3 on T cells, thereby promoting immunological synapse formation, T cell activation, and tumor cell lysis. In addition, the Fc domain extends serum half-life and may contribute to stability. TCE, T cell engager; Fv, variable fragment; scFv, single-chain variable fragment; TAA, tumor-associated antigen; Fab, fragment antigen-binding; Fc, fragment crystallizable; FcR, Fc receptor; TCR, T-cell receptor; ADCC, antibody-dependent cellular cytotoxicity; CDC, complement-dependent cytotoxicity. Created with BioRender.com.

Among immunotherapies, T cell engagers (TCEs) emerged as a promising approach. TCEs are a class of T cell–redirecting therapeutics that promote antitumor activity by bringing T cells into close proximity with malignant cells. These agents encompass a range of engineered platforms that differ in structure, target selection, and mechanisms of immune activation (2). Typical TCEs are bispecific antibodies that connect T cells with tumor cells by simultaneously targeting T cell receptors (TCRs) and tumor-associated antigens (TAAs). Two main structural classes of TCEs exist. The first class, IgG-like TCEs, resembled immunoglobulin G (IgG), consisting of two heavy chains and two light chains with a crystallizable fragment (Fc) domain and two antigen-binding fragments (Fab), each containing one variable fragments (Fv) (3). Unlike natural antibodies, whose Fvs bind the same antigen, IgG-like TCEs had one Fv targeting the TAA and the other binding TCR complex, usually CD3 molecules. The presence of an Fc domain can confer additional effector functions, such as antibody-dependent cell-mediated cytotoxicity (ADCC) and complement-dependent cytotoxicity (CDC) (4). However, in CD3-targeting TCEs, an active Fc domain is not universally beneficial. Fcγ receptor (FcγR)-mediated cross-linking by myeloid cells may cause tumor-independent clustering of the CD3-binding arm, leading to off-tumor T cell activation, systemic cytokine release, and heightened toxicity. Moreover, FcγR engagement can trigger ADCC or antibody-dependent cellular phagocytosis (ADCP) against TCE-bound T cells, thereby depleting the very effector cells required for antitumor activity. Consequently, managing Fc-mediated liabilities is a key consideration in TCE design. To address these issues, many contemporary TCE formats incorporate Fc-silencing or effector-null mutations to retain favorable pharmacokinetic properties while minimizing FcγR-related adverse effects. The second class, Fv-based TCEs, featured a simpler design, usually consisting only of single-chain variable fragments (scFv) formed by artificial fusion of the variable regions of heavy and light chains (5). Their small size facilitated stable cytolytic synapses and improved tumor penetration. While IgG-like TCEs can be challenging to produce due to chain mispairing, Fv-based TCEs suffer from short half-lives, often necessitating frequent dosing. Although both TCE classes present distinct challenges related to stability, half-life, and safety, ongoing engineering of the Fc domain and exploration of novel multispecific architectures are expected to yield improved next-generation TCE designs (**Figure 1**).

TCEs bound TAAs at one end and CD3 molecules on T cells at the other, forming physical connections that induced immune synapses. This interaction activated T cells, triggering release of cytotoxic molecules such as perforin and granzyme to directly induce tumor cell apoptosis (6). Simultaneously, TCEs promoted T cell proliferation and cytokine secretion, including IL-2 and IFN-γ, enhancing antitumor responses (7). Moreover, some IgG-like TCEs retain an Fc domain that may support ADCC and ADCP. Nevertheless, these potential benefits must be carefully balanced against FcγR-mediated risks.

The best-known TCE was blinatumomab, a bispecific antibody targeting CD19 and CD3, which the FDA approved in 2017 for the treatment of relapsed/refractory B-cell acute lymphoblastic leukemia (B-ALL) (8). The FDA approval of blinatumomab confirmed the significance of TCEs in treating hematologic malignancies.

Building on the success of TCEs in hematologic malignancies, increasing numbers of trials investigated their potential use in solid tumors. However, unlike in hematologic cancers, TCE application in solid tumors faced several limitations. Solid tumors typically possessed an immunosuppressive tumor microenvironment (TME), including physical barriers such as dense stroma and hypoxia, as well as immune-suppressive cells like regulatory T cells (Tregs) and myeloid-derived suppressor cells (MDSCs) (9). Additionally, solid tumors often lacked specific antigens, increasing the risk of on-target, off-tumor toxicity and further restricting clinical use (10).

With advances in TCE engineering, including the development of trispecific antibodies and conditional activation techniques, overcoming these challenges in solid tumors became promising (11). Notably, tarlatamab (AMG 757) received accelerated FDA approval in May 2024 for the treatment of adults with extensive-stage small cell lung cancer (ES-SCLC) who experienced disease progression on or after platinum-based chemotherapy (12).

This review summarized the latest clinical trials and data, exploring the clinical potential of TCEs in solid tumors. Furthermore, it discussed the challenges of TCE application and innovative strategies to address these obstacles, such as trispecific antibodies, conditional activation techniques, and combination therapies with other antitumor agents. In parallel, we discuss the identification and application of predictive biomarkers, which are critical for patient stratification and monitoring of acquired resistance. Although the primary focus is on TCEs, we also briefly cover other immune cell engagers, including natural killer (NK) cell engagers, macrophage engagers, and immune cell engagers, to place engager-based cancer immunotherapy within a broader context.

## 2 Breakthrough of TCE in solid tumor

Clinical trials of TCEs in solid tumors have made significant progress in recent years, particularly in metastatic uveal melanoma (mUM) and small cell lung cancer (SCLC). Here, we highlight several landmark clinical trials and their outcomes to evaluate the efficacy of TCEs in solid tumors.

### 2.1 Tebentafusp: the first bispecific antibody TCE for solid tumors

Tebentafusp represented a revolutionary advancement in immunotherapy as the first TCE approved for solid tumors (13). Structurally, tebentafusp is an ImmTAC molecule consisting of a high-affinity soluble TCR linked to an anti-CD3 scFv. Unlike conventional antibody-based TCEs that target surface TAAs, tebentafusp recognizes an intracellular tumor-associated peptide, gp100, presented in the context of HLA-A*02:01. By simultaneously binding the gp100–HLA complex on tumor cells and CD3 on T cells, tebentafusp mediates T cell recruitment and activation, thereby promoting targeted antitumor cytotoxicity (14).

Clinically, tebentafusp demonstrated remarkable efficacy across multiple trials. In the Phase II IMCgp100-102 study (NCT02570308), heavily pretreated mUM patients achieved a 1-year overall survival (OS) rate of 62% and a median OS of 16.8 months, significantly surpassing historical benchmarks. Long-term follow-up (median 48.5 months) revealed durable responses, with a 4-year OS rate of 14%, underscoring its potential for sustained disease control (15). The subsequent Phase III IMCgp100-202 trial (NCT03070392) further validated these findings in treatment-naïve mUM, where tebentafusp reduced the risk of death by 49% compared to investigator’s choice therapies (such as pembrolizumab, ipilimumab, or dacarbazine) (16). Notably, the 1-year OS was 73.2% (vs. 58.5% in controls), and progression-free survival (PFS) improved significantly (HR = 0.73), despite modest response rates. This apparent disconnect stems from tebentafusp’s unique mechanism of action. As a TCR/anti-CD3 fusion protein, tebentafusp redirects T cells against the gp100 peptide–HLA complex, inducing not only direct tumor killing but also broader immune activation and remodeling of the TME (17). These effects may enable prolonged control of residual or occult disease, even in the absence of marked radiographic tumor regression, a pattern distinct from that of cytotoxic agents and many targeted therapies. The clinical profile of tebentafusp thus suggests that the benefit of TCEs in solid tumors may not be fully captured by tumor shrinkage alone; rather, sustained immune-mediated disease control likely underlies the observed survival advantage. Safety data indicated that cytokine release syndrome (CRS)—manifesting as fever, rash, and pruritus—was the most common adverse event, though typically grade 1–2 and manageable with protocolized dosing.

Beyond its established role in mUM, ongoing trials explored tebentafusp’s broader applicability, including neoadjuvant use in locally advanced UM (NCT06414590), combinations with anti-PD-1 agents like pembrolizumab (NCT05549297), and directed therapy of minimal residual disease (NCT05315258) to prevent relapse (18, 19).

The success of tebentafusp marked a paradigm shift in solid tumor immunotherapy, proving that TCEs could deliver meaningful survival benefits even in aggressive, immunotherapy-resistant cancers. Future research would focus on expanding its use to other solid tumors and optimizing combinational strategies to amplify antitumor activity. As the first clinically validated TCE for solid tumors, tebentafusp paved the way for a new generation of immunotherapies. Moreover, its efficacy pattern indicates that survival benefit may occur despite low objective response rates, suggesting that conventional endpoints such as ORR may underestimate the therapeutic impact of immune-redirection strategies. More broadly, tebentafusp supports the notion that future evaluation of TCEs in solid tumors should incorporate not only radiographic endpoints but also additional indicators, such as immune functional status and biomarker assessments.

### 2.2 Tarlatamab: the breakthrough of TCE for solid tumors

SCLC is an aggressive neuroendocrine tumor subtype with limited treatment options, especially for relapsed extensive-stage disease. Although lurbinectedin showed promising activity and received accelerated FDA approval based on phase 2 data, subsequent phase 3 results failed to demonstrate an OS benefit, and no standard backline therapy has yet been established (20, 21). Tarlatamab has offered new hope for this patient population.

Delta-like ligand 3 (DLL3), an inhibitory ligand of the Notch signaling pathway, promotes tumorigenesis in SCLC by suppressing Notch-mediated differentiation and driving uncontrolled proliferation of neuroendocrine cells (22). DLL3 is specifically expressed on the surface of SCLC tumor cells and is minimally expressed in normal adult tissues (23). Thus, DLL3 is considered an ideal therapeutic target for SCLC.

Tarlatamab is a bispecific antibody that binds both DLL3 on SCLC tumor cells and CD3 on T cells (24). This dual binding facilitates immune synapse formation between T cells and tumor cells, activating T cell–mediated tumor lysis (25). T cells engaged with tumor cells release cytotoxic granules containing perforin and granzyme, inducing apoptosis of DLL3-expressing cancer cells. Concurrently, this interaction triggers secretion of pro-inflammatory cytokines, such as IFN-γ and TNF-α, enhancing the antitumor immune response and promoting bystander effect (26).

Unlike typical bispecific antibodies, tarlatamab is a Half-life extended BiTE (HLE-BiTE), containing an IgG Fc domain integrated into its core BiTE structure (27). The Fc domain confers several pharmacokinetic and pharmacodynamic advantages. By avoiding lysosomal degradation and enabling recycling back into systemic circulation, the Fc domain extends tarlatamab’s serum half-life (28). This extended half-life allows less frequent dosing—biweekly administration instead of daily or weekly regimens typical of earlier BiTEs—improving treatment adherence and reducing logistical burdens for patients (**Figure 1**). During the dose-expansion phase, tarlatamab demonstrated encouraging antitumor activity in relapsed ES-SCLC, achieving an objective response rate (ORR) of 23.4% (95% confidence interval [CI], 15.7 to 32.5) and a disease control rate (DCR) of 51.4% (95% CI, 41.5 to 61.2) (29). An increasing number of clinical trials evaluating its efficacy and safety are underway.

The DeLLphi-300 trial evaluated whether tarlatamab could provide long-term benefits for patients with ES-SCLC (30). The study enrolled 152 patients treated with tarlatamab, with a median follow-up of 12.1 months (range, 0.2 to 34.3). It conclusively demonstrated that tarlatamab exhibited sustained antitumor activity and clinically meaningful survival benefits in ES-SCLC, with a pronounced therapeutic advantage in the 10 mg dose cohort. The 10 mg group achieved an mOS of 20.3 months and an ORR of 35.3%, outperforming both the higher dose group and historical benchmarks for second-line therapies, where mOS rarely exceeded 9 months. The median duration of response (DoR) was 11.2 months across all cohorts, underscoring the durability of response and highlighting tarlatamab’s ability to slow disease progression. No new safety signals were detected. The trial showed unprecedented survival outcomes in previously treated SCLC. Additionally, results suggested tarlatamab’s efficacy against brain metastases in SCLC. Among patients with baseline central nervous system (CNS) lesions ≥10 mm—including those with tumor shrinkage long after prior brain radiotherapy—62.5% (10 of 16) experienced CNS tumor shrinkage of at least 30%. Brain metastasis is a common and challenging complication of SCLC, and this finding offers a novel approach to managing this condition.

The DeLLphi-301 trial, a multicenter phase 2 study, comprehensively assessed the antitumor efficacy, safety profile, adverse event spectrum, and pharmacokinetics of tarlatamab in patients with relapsed ES-SCLC (31). The study enrolled 220 patients who had previously received multiple lines of therapy and administered tarlatamab intravenously every two weeks at doses of 10 mg or 100 mg, with stratification based on prior platinum sensitivity and baseline CNS involvement. Median follow-up durations were 10.6 months for the 10 mg cohort and 10.3 months for the 100 mg cohort. The mPFS was 4.9 months (95% CI, 2.9–6.7) in the 10 mg group and 3.9 months (95% CI, 2.6–4.4) in the 100 mg group. Nine-month overall survival rates were 68% and 66%, respectively. ORRs were 40% (97.5% CI, 29–52) in the 10 mg group and 32% (97.5% CI, 21–44) in the 100 mg group. These results suggested that dose escalation did not confer a survival benefit. Regarding safety, the most frequent adverse events included CRS (51% in the 10 mg group and 61% in the 100 mg group), reduced appetite (29% and 44%, respectively), and fever (35% and 33%, respectively). CRS primarily occurred during the first treatment cycle, with most cases being grade 1 or 2 in severity. Grade 3 CRS was less common in the 10 mg group (1%) compared to the 100 mg group (6%). Only 3% of patients discontinued tarlatamab due to treatment-related adverse events (TrAEs). The study demonstrated tarlatamab’s antitumor activity, durable objective responses, and promising survival outcomes in patients with previously treated SCLC. In May 2024, the FDA approved tarlatamab for treatment of ES-SCLC in later-line settings (12).

Additionally, the trial revealed an unexpected inverse relationship between dose intensity and clinical efficacy. The 10 mg dose appeared optimal, balancing good efficacy with a favorable safety profile. This inverse dose-response relationship suggests that the optimal dose of a TCE may be determined by immune biology rather than by maximal drug exposure (32). Because tarlatamab exerts its activity through DLL3-directed T-cell redirection, its efficacy depends on forming sufficient immune synapses and sustaining T-cell function, not simply on increasing drug concentration. Higher doses may trigger excessive cytokine release and immune overactivation, leading to greater toxicity and potentially causing early T-cell dysfunction or exhaustion. These findings highlight the need to define an optimal biological dose rather than relying solely on the traditional maximum tolerated dose.

Phase I and II trials demonstrated tarlatamab’s efficacy and safety in later-line ES-SCLC settings. FDA approval of tarlatamab represents a significant breakthrough for TCE application in solid tumors. Future phase III trials are needed to confirm its efficacy and safety and to explore its long-term benefits. Research directions include optimizing the standardized treatment dose, identifying predictive efficacy markers, investigating combination strategies with immune checkpoint inhibitors (ICIs), and validating the intracranial efficacy observed in subgroup analyses.

### 2.3 TCEs targeting other TAAs: currently undergoing clinical trials

The search for suitable TAAs remains a fundamental challenge in developing TCEs. Ideal targets should exhibit high tumor specificity and stable expression to minimize the risk of on-target, off-tumor toxicity while maximizing antitumor efficacy. To date, only a limited number of TCEs, such as those targeting gp100 and DLL3, have received FDA approval.

However, the field is rapidly expanding, with numerous novel TCEs directed against other promising TAAs currently undergoing clinical evaluation. As summarized in **Table 1**, several investigational TCEs are being tested in clinical trials targeting antigens such as CLDN6 (expressed in ovarian and testicular cancers), EpCAM (common in gastrointestinal and breast cancers), MUC16 (a marker for ovarian cancer), HER2 (overexpressed in breast and gastric cancers), PSMA (a prostate cancer antigen), and others. These TCEs aim to address unmet medical needs, particularly in relapsed or refractory cancers where conventional therapies have failed.

**Table 1:** All clinical trials of TCEs in solid tumor (July 2025).

Agent	Format	Platform	Additional target/domain	Indications	Trial; phase	Trial status	Results	Refs.
Ubamatamab (REGN4018)	MUC16 x CD3 IgG4 bispecific antibody	IgG4-based TCE	N/A	Ovarian Cancer	NCT03564340;Phase II NCT06787612;Phase II	Recruiting	Ubamatamab monotherapy demonstrated a 14.3% ORR and manageable safety; ubamatamab plus cemiplimab achieved an ORR of 18.2% with manageable safety.	(33, 34)
AMG910	CLDN18.2 x CD3 scFv-based bispecific antibody	HLE BiTE^®^	Fc	Claudin 18.2-positive G/GEJ adenocarcinoma	NCT04260191;Phase I	Terminated	N/A	(35)
AZD5863	CLDN18.2 x CD3 VHH-based 2 + 1 bispecific antibody	VHH-based TCE	Fc (silenced)	Advanced or metastatic solid tumors	NCT06005493;Phase I/II	Recruiting	N/A	(36)
IBI389	CLDN18.2 x CD3 asymmetric 2 + 1 IgG-based bispecific antibody	2 + 1 TCE	Fc	G/GEJC;PDAC	NCT05164458;Phase I	Recruiting	IBI389 demonstrated a 22.2% ORR and manageable safety in patients with CLDN18.2-positive G/GEJC.	(37)
ASP2138	CLDN18.2 x CD3 asymmetric 2 + 1 IgG-based bispecific antibody	2 + 1 TCE	Fc	CLDN 18.2-positive advanced G/GEJC and PDAC	NCT05365581;Phase I	Recruiting	N/A	(38)
Cibisatamab (RO6958688)	CEA x CD3 asymmetric 2 + 1 IgG1 bispecific antibody	TCB	Fc	CEA-positive solid tumors	NCT03337698;Phase I/II	Completed	Cibisatamab monotherapy demonstrated a 7% ORR (median DOR 4.3 months) in MSS colorectal cancer with manageable safety; cibisatamab plus atezolizumab achieved an 18% ORR (median DOR 11.8 months) with manageable safety.	(39)
ERY974	GPC3 x CD3 IgG4 bispecific antibody	ART-Ig	Fc	Locally advanced or metastatic HCC	NCT05022927;Phase I	Active, not recruiting	N/A	(40)
SAR-4442000	GPC3 x TCRαβ multi-valent VHH-based bispecific antibody	Nanobody^®^	anti-albumin VHH	Advanced solid tumors	NCT05450562;Phase I/II	Terminated	SAR444200 demonstrated no DLTs and manageable safety (CRS 62%, all grade 1-2) in the first two dose levels (1-3 mg).	(41)
JANX008	EGFR x CD3 trispecific TCE	TRACTr	anti-albumin VHH, dual masks	Advanced or metastatic solid tumor malignancies	NCT05783622;Phase I	Recruiting	JANX008 demonstrated encouraging efficacy (1 confirmed PR with 100% target lesion regression) and manageable safety (grade 1 CRS only, no DLTs).	(42)
CX-904	EGFR x CD3 bispecific IgG prodrug	Probody^®^	Masking peptide	Advanced solid tumors	NCT05387265;Phase I	Terminated	CX-904 monotherapy demonstrated a 33% ORR in pancreatic cancer (2/6 confirmed PRs, 100% disease control) and a favorable safety profile (no grade ≥2 CRS) in heavily pretreated advanced solid tumors.	(43)
CC-3	B7-H3 x CD3 symmetric 2 + 2 IgG-based bispecific antibody	IgG-based TCE	Fc	Metastatic CRC	NCT05999396;Phase I	Recruiting	N/A	(44)
XmAb-819	ENPP3 x CD3 2 + 1 bispecific antibody	XmAb^®^ 2 + 1	Fc	Advanced solid tumors	NCT05433142;Phase I	Recruiting	XmAb819 monotherapy demonstrated a 25% ORR (70% DCR) and manageable safety in heavily pretreated advanced clear cell renal cell carcinoma.	(45)
JNJ-79032421	MSLN x CD3 symmetric 2 + 2 bispecific antibody	Membrane-restricted T-cell engager	Fc	Advanced solid tumors	NCT06255665;Phase I	Completed	N/A	(46)
Brenetafusp (IMC-F106C)	PRAME x TCR scFv-TCR fusion bispecific antibody	ImmTAC^®^	N/A	Treatment naive HLA-A*02:01-positive advanced melanoma	NCT06112314;Phase III	Recruiting	IMC-F106C monotherapy demonstrated a 61% clinical benefit rate (65% in PRAME-positive patients) and manageable safety in checkpoint-pretreated advanced melanoma.	(47)
AMV564	CD33 x CD3 tetravalent tandem diabody	TandAb	N/A	Advanced solid tumors	NCT04128423;Phase I	Unknown status	N/A	(48)
Xaluritamig (AMG 509)	STEAP1 x CD3 2 + 1 bispecific antibody	XmAb^®^ 2 + 1	Fc	mCRPC	NCT06555796;Phase I	Active, not recruiting	N/A	(49)
AMG 199	MUC17 x CD3 scFv-based bispecific antibody	HLE BiTE^®^	Fc	GC/GEJC	NCT04117958;Phase I	Terminated	N/A	(50)
XmAb541	CLDN6 x CD3 2 + 1 bispecific antibody	XmAb^®^ 2 + 1	Fc	GCT and other CLDN6+ solid tumors	NCT06276491;Phase I	Recruiting	N/A	(51)
Solitomab (AMG110)	EpCAM x CD3 scFv-based bispecific antibody	BiTE^®^	N/A	Advanced adenocarcinoma	NCT00635596;Phase I	Completed; Results unavailable	MT110 monotherapy demonstrated 38% stable disease rate (median duration 155 days) and manageable toxicity with step-dose and dexamethasone; no RECIST responses were observed.	(52)
Runimotamab (RG6194)	HER2 x CD3 bispecific antibody	TDB	Fc	Breast Cancer	NCT03448042;Phase I	Recruiting	Runimotamab monotherapy demonstrated a 78% grade 1-2 CRS rate; runimotamab plus trastuzumab achieved a 30.4% ORR with lower CRS (31%, grade 1-2) and improved tolerability in heavily pretreated HER2-positive breast cancer.	(53)
AMG 340	PSMA x CD3 scFv-based bispecific antibody	HLE BiTE^®^	Fc	mCRPC	NCT04740034;Phase I	Terminated	AMG 340 demonstrated manageable CRS (52%, grade ≥3 2%) with low-affinity CD3 design, but no RECIST responses were observed, and the study was discontinued due to insufficient clinical activity.	(54)
JNJ-081	PSMA x CD3 IgG4-PAA bispecific antibody	IgG4-based T-cell engager	Fc	Advanced solid tumors	NCT03926013;Phase I	Completed	JNJ-081 demonstrated transient PSA declines but no radiographic responses; CRS was partially mitigated by SC dosing and step-up priming; ADA formation limited drug exposure.	(55)
AMG160	PSMA x CD3 scFv-based bispecific antibody	HLE BiTE^®^	Fc	mCRPC	NCT03792841;Phase I	Terminated	N/A	(56)

TCE, T cell engager; ORR, objective response rate; DCR, disease control rate; CRS, cytokine release syndrome; DLT, dose-limiting toxicity; PR, partial response; SD, stable disease; G/GEJ, gastric/gastroesophageal junction; HCC, hepatocellular carcinoma; mCRPC, metastatic castration-resistant prostate cancer; CRC, colorectal cancer; PDAC, pancreatic ductal adenocarcinoma; MSS, microsatellite stable; HLE, half-life extended; BiTE, bispecific T cell engager; ImmTAC, immune mobilizing monoclonal T cell receptor against cancer; TRACTr, tumor-activated T cell engager; ADA, anti-drug antibody. Collected from https://clinicaltrials.gov/; accessed on1 July 2025. Reported efficacy and safety data are based on interim or final results where available.3 Challenges of the application in solid tumors.

The future of TCE therapy depends on the discovery and validation of additional novel TAAs. Advances in genomics, proteomics, and bioinformatics are accelerating the identification of TAAs with optimal pharmacological properties. As research progresses, more TCEs targeting diverse TAAs are expected to enter clinical trials, broadening treatment options for patients with refractory cancers and potentially transforming the oncology therapeutic landscape.

## 3 Challenges of the application in solid tumors

### 3.1 Immunosuppression of the TME

**Figure 2:**
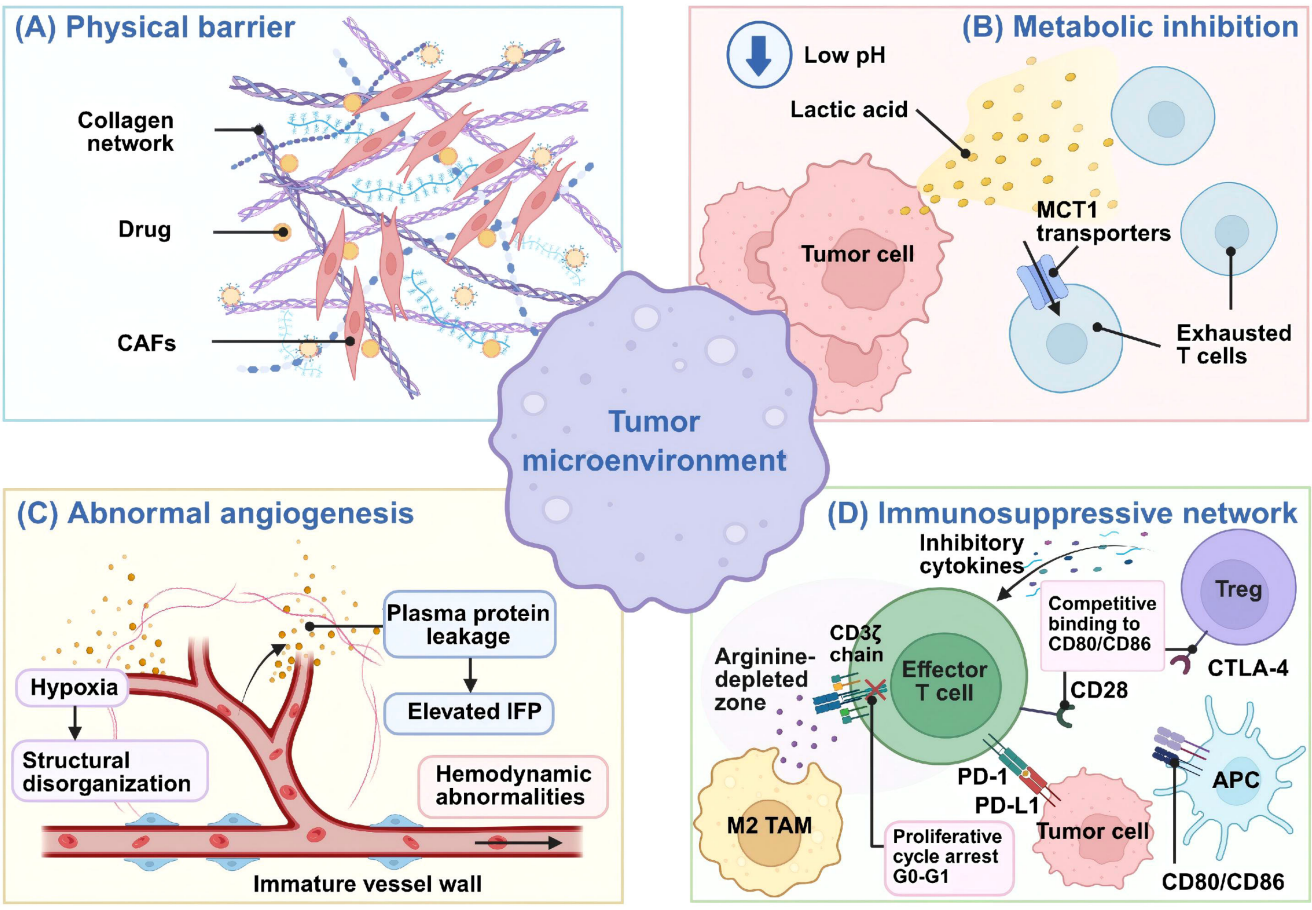
Major barriers within the tumor microenvironment that limit antitumor immunity and therapeutic efficacy. Barriers in the TME that restrict drug delivery and impair antitumor immunity. **(A)** Physical barrier: a dense, collagen-rich extracellular matrix and CAFs impede therapeutic penetration. **(B)** Metabolic inhibition: low pH and lactic acid, together with MCT1-mediated lactate transport, promote T-cell dysfunction and exhaustion. **(C)** Abnormal angiogenesis: immature, disorganized, and leaky vessels lead to hypoxia, plasma protein leakage, elevated IFP, and hemodynamic abnormalities. **(D)** Immunosuppressive network: Tregs, M2 TAMs, and APCs release inhibitory cytokines and deplete nutrients, while the checkpoint axes PD-1/PD-L1 and CTLA-4, competing with CD28 for CD80/CD86, arrest effector T cells in G0–G1. CAF, cancer-associated fibroblast; ECM, extracellular matrix; TAM, tumor-associated macrophage; APC, antigen-presenting cell; Treg, regulatory T cell; IFP, interstitial fluid pressure; MCT1, monocarboxylate transporter 1. Created with BioRender.com.

Unlike circulating or anatomically accessible tumor cells, solid tumors feature a highly organized and heterogeneous microenvironment that disrupts multiple steps essential for TCE activity, including tumor accumulation, stromal penetration, effector T cell recruitment, immune synapse formation, and sustained cytotoxicity. Key barriers include a dense extracellular matrix (ECM), metabolic suppression, immunosuppressive networks, and abnormal angiogenesis (**Figure 2**).

#### 3.1.1 ECM and stromal barriers limit intratumoral TCE distribution

The ECM represents a major structural obstacle in solid tumors. Its dense, dysregulated composition, comprising collagen, fibronectin, proteoglycans, and hyaluronic acid, restricts molecular diffusion, increases tissue stiffness, and elevates interstitial pressure (57). These properties broadly hinder drug delivery and are especially detrimental to TCEs, as their efficacy depends not only on tumor penetration by the engager itself but also on the infiltration of effector T cells.

In this setting, excessive stromal deposition can restrict intratumoral diffusion of TCEs, leading to heterogeneous drug exposure across the tumor mass. Concurrently, stromal compression and matrix remodeling impede T cell infiltration, thereby reducing contact between activated T cells and tumor cells and compromising TCE-mediated immune synapse formation (58, 59). Cancer-associated fibroblasts (CAFs) further strengthen this barrier by promoting fibrosis, matrix remodeling, and immune exclusion (60). As a result, stromal constraints in solid tumors may attenuate TCE efficacy even when systemic drug levels are adequate, potentially explaining the higher doses sometimes needed to achieve antitumor activity, and the associated increase in off-tumor toxicity risk.

#### 3.1.2 Metabolic dysregulation compromises T cell function

Beyond physical exclusion, the metabolically hostile TME imposes a major functional constraint on TCE therapy. Because TCEs do not kill tumor cells directly but instead redirect T cells to mediate cytotoxicity, their efficacy depends on the metabolic fitness of intratumoral T cells. However, solid tumors commonly exhibit hypoxia, glucose deprivation, lactate accumulation, and extracellular acidosis, conditions well established to suppress T cell proliferation, cytokine secretion, persistence, and cytolytic activity (61).

These metabolic constraints directly impair TCE pharmacodynamics. Even when a TCE successfully bridges CD3 on T cells with a TAA on tumor cells, the resulting activation signal may fail to elicit durable or effective antitumor responses under metabolically unfavorable conditions. T cells exposed to such stress often show impaired expansion, reduced release of effector molecules, and diminished serial killing capacity. Thus, metabolic suppression not only defines the general nature of the TME but also specifically undermines the cellular effector machinery that TCEs rely on (62).

#### 3.1.3 Immunosuppressive networks attenuate TCE-mediated immune activation

Another critical limitation stems from the immunosuppressive network present in solid tumors. Tregs, myeloid-derived suppressor cells, tumor-associated macrophages (TAMs), and suppressive mediators such as TGF-β, IL-10, and adenosine collectively dampen antitumor immunity and promote T cell dysfunction (63). Although TCEs can bypass MHC restriction and forcibly redirect T cells toward tumor cells, they cannot fully overcome the inhibitory signals that constrain T cell activation, expansion, and persistence within the TME.

This issue is especially pronounced in poorly inflamed or “cold” tumors, where baseline infiltration of functional CD8^+^ T cells is limited (64). Under these conditions, the pool of recruitable effector T cells is insufficient, thereby restricting TCE activity from the outset. Moreover, checkpoint signaling and exposure to suppressive cytokines can drive T cell exhaustion, further blunting the cytotoxic response triggered by TCE-mediated engagement (65). These findings help explain why TCE efficacy often depends on the immune contexture of the tumor and why combination strategies with ICIs or costimulatory approaches may be required to restore T cell responsiveness and improve therapeutic outcomes.

#### 3.1.4 Abnormal vasculature impairs TCE delivery and T cell trafficking

Abnormal angiogenesis poses an additional barrier to TCE efficacy in solid tumors. Tumor vasculature is often disorganized, compressed, and hyperpermeable, leading to poor perfusion, elevated interstitial fluid pressure (IFP), and regional hypoxia (66, 67). For TCEs, these vascular abnormalities impair both intratumoral drug delivery and immune cell trafficking.

From a pharmacologic standpoint, dysfunctional vasculature limits TCE extravasation and results in uneven distribution within tumor tissue, particularly in poorly perfused regions. This spatial heterogeneity may prevent TCEs from reaching adequate local concentrations at sites harboring tumor cells. Concurrently, abnormal vascular architecture and endothelial dysfunction hinder T cell transmigration and infiltration into the tumor (68). Consequently, the two essential components of TCE activity, the engager and the effector T cell, may fail to co-localize in sufficient quantities within the same intratumoral niches.

### 3.2 On-target, off-tumor toxicity

In tumor immunotherapy, on-target, off-tumor toxicity remains a major challenge limiting the efficacy and safety of antitumor drugs (69). Because therapeutic targets are often co-expressed in normal tissues, immune cells or drugs cannot distinguish tumor cells from normal cells, leading to toxicity in healthy tissues (70). Ideal targets should exhibit high tumor specificity, minimal expression in normal tissues, and strong binding affinity. However, most targets are TAAs that are overexpressed in tumors but not entirely tumor-specific (71). Compared to hematologic malignancies, the heterogeneity of solid tumors intensifies this challenge. Expression of the same target may vary significantly across tumor subtypes or microenvironments (72). Moreover, in hematologic malignancies, tumor cells circulate in the blood, allowing drugs or CAR-T cells to directly access target cells. In contrast, physical barriers and a complex immune microenvironment in solid tumors necessitate higher drug doses to achieve efficacy, increasing the risk of off-tumor toxicity (73).

### 3.3 Mechanisms of primary and acquired resistance to TCEs in solid tumors

The efficacy of TCEs in solid tumors remains limited by multiple mechanisms of primary and acquired resistance. These mechanisms may arise from tumor-intrinsic alterations, defects in antigen presentation, T cell dysfunction, and adaptive immunosuppression. A deeper understanding of these resistance pathways is essential for improving patient selection and designing more effective combination strategies.

#### 3.3.1 Antigen loss or downregulation

Antigen loss or downregulation represents a central mechanism of acquired resistance to TCEs. Because TCE-mediated antitumor activity strictly depends on sufficient target density at the tumor cell surface, therapeutic exposure can exert selective pressure that favors the outgrowth of pre-existing antigen-low or antigen-negative subclones (74). Concurrently, tumor cells may actively reduce antigen levels through adaptive transcriptional reprogramming, epigenetic silencing, altered membrane trafficking, enhanced internalization, or proteolytic shedding of the target molecule (75). Reduced antigen density directly impairs TCE pharmacodynamic activity by limiting target engagement and weakening the physical bridge required to bring effector T cells into contact with tumor cells. This, in turn, disrupts immune synapse assembly and decreases the likelihood that TCR/CD3 signaling will reach the threshold needed to sustain T cell activation, cytotoxic granule polarization, and serial killing (74). In solid tumors, where baseline target expression is frequently heterogeneous, even modest antigen downregulation may be sufficient to push tumor cells below the functional threshold, enabling immune escape (76, 77). This resistance mechanism resembles antigen escape observed with other targeted immunotherapies but may be especially critical for TCEs, as their efficacy relies on continuous, spatially localized, and quantitatively adequate target expression to maintain T cell–tumor cell engagement. Collectively, these observations indicate that antigen instability is not merely associated with treatment failure but actively drives relapse and progressive loss of response during TCE therapy.

#### 3.3.2 Low or heterogeneous target antigen expression

In addition to antigen loss during treatment, low baseline expression or marked intratumoral heterogeneity of the target antigen can contribute to primary resistance. Unlike hematologic malignancies, many solid tumors do not uniformly express a given surface antigen across all tumor cells (78). Consequently, TCEs may eliminate only antigen-high subpopulations while sparing antigen-low or antigen-negative cells, enabling residual disease to persist and progress. Heterogeneous expression can also limit the bystander effect and reduce both the depth and durability of the response (79). These observations underscore the importance of selecting targets with sufficiently high and homogeneous tumor expression for TCE development in solid tumors.

#### 3.3.3 MHC-I downregulation and impaired antigen presentation

For TCE platforms that rely on peptide–HLA recognition, such as ImmTAC-type constructs, resistance may also arise from defects in MHC-I expression and antigen presentation. Mechanistically, MHC-I downregulation can result from immune editing under therapeutic pressure or from tumor-intrinsic alterations, including genetic loss of HLA alleles or β2-microglobulin (B2M), epigenetic silencing of HLA-related genes, impaired IFN-γ signaling, or transcriptional reprogramming associated with tumor dedifferentiation (80). In parallel, abnormalities in the antigen-processing machinery, such as defective proteasomal processing, reduced TAP-mediated peptide transport, or impaired peptide loading and HLA complex assembly, can further diminish the surface display of relevant peptide–HLA complexes. These defects reduce both the density and stability of targetable peptide–MHC-I complexes, thereby weakening therapeutic efficacy, impairing immune synapse formation, and diminishing downstream T cell activation and cytotoxicity (81). Because ImmTAC-like molecules depend on accurate presentation of intracellularly derived peptides rather than surface antigen abundance alone, disruption of this pathway represents a particularly important mechanism of both primary and acquired resistance (82). Thus, HLA genotype, MHC-I integrity, and the functional status of the antigen-presentation machinery may serve as critical determinants of therapeutic responsiveness in this context.

#### 3.3.4 T cell dysfunction and exhaustion

Even when TAA recognition is preserved, TCE efficacy may still be limited by impaired T cell fitness. Within solid tumors, persistent antigen exposure, chronic inflammatory signaling, metabolic stress, and an immunosuppressive TME can drive T cell dysfunction and exhaustion (83). Exhausted T cells typically exhibit reduced proliferative capacity, diminished cytokine production, impaired cytotoxicity, and sustained expression of inhibitory receptors. Under these conditions, redirected T cells may fail to sustain durable antitumor activity despite successful initial engagement by the TCE. This issue may be especially relevant in heavily pretreated patients or in tumors with chronically suppressed immune environments, where endogenous T cells are already compromised in number or function.

#### 3.3.5 Upregulation of immunosuppressive ligands and pathways

Tumors may also adapt to TCE-mediated immune pressure by upregulating immunosuppressive ligands and activating inhibitory pathways (84). This adaptive response can dampen T cell activation and undermine sustained antitumor immunity. For instance, increased expression of inhibitory checkpoint ligands or induction of suppressive cytokine networks may establish a local microenvironment that restricts T cell function following initial redirection. Additionally, the recruitment or activation of immunosuppressive cell populations can further reinforce this resistant state. Although such adaptive immune escape mechanisms may not completely block initial responses, they can contribute to incomplete tumor regression, rapid disease progression, or eventual treatment failure.

Importantly, these resistance mechanisms rarely act in isolation. Instead, they often coexist and mutually reinforce one another during disease progression and therapy. For example, heterogeneous antigen expression may impair initial tumor clearance, while chronic stimulation from suboptimal T cell responses can subsequently drive exhaustion and adaptive immunosuppression. Concurrently, stromal exclusion may limit adequate T cell infiltration, thereby amplifying the impact of tumor-intrinsic resistance mechanisms. Recognizing the interplay among these pathways has critical implications for the future development of TCEs in solid tumors. Effective therapeutic strategies may need to incorporate more rigorous target selection, dynamic monitoring of antigen expression, assessment of antigen-presentation capacity, and evaluation of baseline T cell competence. Furthermore, rational combination approaches, such as those aimed at reversing T cell exhaustion, counteracting immunosuppressive signaling, or remodeling stromal barriers, may be necessary to enhance the durability of clinical responses.

## 4 Innovative strategies to overcome challenges

Through multidimensional technological breakthroughs, many innovative strategies have been applied to TCE design, offering new hope for their widespread use in treating solid tumors. These pivotal innovations can be categorized into optimized TCE antibody designs, systematic combination therapies, and precise selection of TAAs.

### 4.1 The optimized designs of TCEs

Before discussing next-generation optimization strategies, it is important to consider clinically approved TCEs, which have already provided valuable insights into molecular design. Approved TCEs reflect several distinct protein engineering approaches, each with specific advantages and limitations. Fc-free tandem scFv BiTEs, such as blinatumomab, promote more efficient immune synapse formation but require continuous infusion due to their short half-life (8). In contrast, half-life–extended and IgG-like formats improve pharmacokinetics, stability, and dosing convenience; however, their larger molecular size may limit tumor penetration, and prolonged exposure can raise safety concerns (85). Avidity-enhanced architectures, exemplified by the 2 + 1 format of glofitamab, further improve tumor binding and selectivity, albeit at the cost of increased structural complexity (86). Tebentafusp expands this design space through a TCR–CD3 fusion strategy that targets intracellular peptide–HLA complexes in an HLA-restricted manner (14). Collectively, these approved agents underscore the need to balance potency, drug exposure, tissue access, manufacturability, and safety in next-generation TCE design. The major engineering strategies, along with the advantages and limitations of several approved TCE formats, are summarized in **Table 2**.

**Table 2:** Comparison of approved TCEs engineering strategies.

Drug	Format	Structural features	Molecular weight	Half-life and administration	Advantages	Limitations
Blinatumomab	BiTE	Two tandem scFvs (anti-CD19 + anti-CD3) joined by a flexible glycine-serine linker; single polypeptide chain; no Fc domain.	~54 kDa	~2 hours; requires continuous IV infusion (28-day cycles).	Small size enables close immune synapse formation; high potency at low concentrations; high tissue penetration.	Short half-life; continuous infusion required; higher CRS risk due to rapid T cell activation.
Mosunetuzumab	1 + 1 IgG-like	Full-length IgG1 with “knob-into-hole” heterodimerization; one arm targets CD20 (Fab), the other targets CD3 (Fab); silent Fc (N297G mutation).	~150 kDa	~7-14 days; administered as fixed-duration IV infusion (8 cycles over 6 months).	Fixed-duration dosing; favorable safety profile.	Less tissue penetration than BiTE; potential infusion reactions.
Glofitamab	2 + 1 IgG-like	Two CD20-binding Fabs and one CD3-binding Fab; “knob-into-hole” with CrossMab; silent Fc.	~200 kDa	~7-14 days; administered as fixed-duration IV infusion (12 cycles total).	Enhanced tumor cell binding; high response rates.	More complex manufacturing; fixed-duration may not suit all patients.
Epcoritamab	IgG1-like (DuoBody^®^)	Full-length IgG1 with controlled Fab-arm exchange; CD20 and CD3 Fabs; silent Fc (K322A mutation).	~150 kDa	~14-21days; subcutaneous injection (weekly then every 2 weeks).	Most convenient SC administration; less CRS due to gradual absorption.	Higher injection volume required; local injection site reactions.
Teclistamab	IgG4-based	BCMA and CD3 Fabs; “knob-into-hole” heterodimerization; silent Fc (S228P mutation for stability).	~150 kDa	~7-14 days; IV infusion (step-up dosing to manage CRS).	Effective in heavily pre-treated multiple myeloma; manageable CRS with step-up dosing.	IV administration; limited to BCMA-expressing tumors.
Talquetamab	IgG4-based	GPRC5D targeting (novel antigen); silent Fc; similar design to teclistamab but with different binding domain.	~150 kDa	~7-14 days; SC injection (weekly or every 2 weeks after step-up).	Effective in BCMA-refractory patients; SC convenience.	Unique on-target toxicities (dysgeusia, nail disorders, skin rash) due to GPRC5D expression in normal tissues.
Elranatamab	IgG2-based	BCMA × CD3; silent Fc; similar to teclistamab but with IgG2 backbone and different CD3 affinity.	~150 kDa	~7-14 days; SC injection (step-up dosing).	Alternative option for BCMA-targeted therapy; SC administration.	Slightly lower response rates than teclistamab in some comparisons; similar BCMA-related limitations.
Tarlatamab	HLE-BiTE	Tandem scFv (DLL3 × CD3) fused to an Fc domain (IgG1-derived); retains BiTE’s single-chain architecture.	~100-120 kDa	~7-10 days; IV infusion (step-up dosing: 1 mg → 10 mg → 10 mg every 2 weeks).	Half-life extension; intermittent dosing possible; retains strong T-cell redirecting activity.	Prolonged systemic exposure may increase toxicity risk; larger size may reduce tissue penetration compared with Fc-free BiTEs.
Tebentafusp	TCR-based TCE (ImmTAC)	High-affinity TCR targeting gp100 peptide-HLA complex, fused to anti-CD3 scFv; no Fc domain; unique among approved TCEs.	~75 kDa	~4-6 hours (short half-life); weekly IV infusion.	First TCE for solid tumor (uveal melanoma); expands targetable antigen space to intracellular proteins.	HLA-restricted (HLA-A*02:01 only); short half-life; significant CRS and skin toxicity.

TCE, T-cell engager; scFv, single-chain variable fragment; BiTE, bispecific T-cell engager; TCR, T-cell receptor; HLA, human leukocyte antigen; BCMA, B-cell maturation antigen; CRS, cytokine release syndrome; SC, subcutaneous; IV, intravenous. Molecular weight and half-life are approximate values and may vary depending on molecular design and clinical dosing context4.1.1 Tri-specific.

#### 4.1.1 Tri-specific

TCE formats have evolved from conventional 1 + 1 bispecific designs to more sophisticated 2 + 1 and 1 + 1 + 1 trispecific formats to enhance potency, selectivity, and resistance control. The monovalent tumor binding of classical 1 + 1 constructs can create a trade-off between efficacy and specificity. In contrast, the 2 + 1 format incorporates two identical tumor antigen–binding domains and one CD3-binding domain, thereby strengthening avidity-dependent recognition of antigen-high tumor cells while minimizing off-tumor activity against cells with low antigen expression (87). Trispecific 1 + 1 + 1 TCEs further extend this concept by either simultaneously targeting two distinct tumor antigens to address heterogeneity and antigen escape, or incorporating a costimulatory receptor, such as CD28, 4-1BB, or OX40, to boost T cell activation, persistence, and antitumor function, or adding half-life–extending moieties like human serum albumin (HSA) to achieve enhanced and sustained immune responses (88).

By incorporating HSA binding domain into the tri-specific antibody, the half-life can be significantly extended (88). This allows biweekly dosing, greatly improving patient compliance—a key strategy for durable immunotherapy in solid tumors (89). HPN328 is a representative TriTAC targeting TAA, CD3, and HSA. Its molecular design features a tri-specific architecture comprising an anti-DLL3 scFv, an anti-CD3 scFv, and an HSA binding domain (90). Binding to HSA extends HPN328’s half-life to 78–187 hours. Additionally, with a molecular weight of approximately 53 kDa—about one-third that of conventional IgG—it demonstrates superior solid tumor penetration, achieving a threefold increase in tumor infiltration in preclinical models compared to IgG-based dual antibodies. This property suggests potential to cross the blood-brain barrier and treat brain metastases. HPN328 is currently under clinical evaluation (91).

TriTCEs targeting TAA, CD3, and co-stimulatory receptors (such as CD28 or CD137) represent an innovative immunotherapeutic class that combines tumor targeting, T cell activation via CD3, and enhanced co-stimulatory signaling through modular design to achieve synergistic antitumor effects (92). These molecules typically adopt a heterodimeric architecture, incorporating a low-affinity CD3-binding domain to reduce CRS risk and a high-affinity TAA-binding domain to ensure preferential localization within the tumor tissue. Co-stimulatory domains further promote T cell expansion, survival, and persistence (93). This design overcomes the limitations of traditional bispecific antibodies by reversing the immunosuppressive microenvironment and demonstrating durable antitumor activity in preclinical models. Representative agents such as Zymeworks’ CLDN18.2 TriTCE and DLL3 TriTCE, as well as Sanofi’ s SAR442257 (CD38/CD3/CD28) and SAR443216 (HER2/CD3/CD28), have entered clinical development, showing promise in gastric cancer, multiple myeloma, and other indications by balancing efficacy and safety (94–96).

Beyond TriTCEs integrating co-stimulatory receptors and HSA-binding domains, some TriTCEs incorporate ICIs to enhance efficacy in solid tumors. These TriTCEs activate T cells while alleviating microenvironmental inhibition—for example, TriTCE ICIs targeting CD3/TAA/PD-L1. Zymeworks’ TriTCE ICI notably reversed T cell exhaustion in a human peripheral blood mononuclear cell transplanted xenograft model (97).

TriTCEs can also be designed to target two distinct tumor antigens in addition to CD3, with the aim of improving recognition of heterogeneous tumors and reducing the risk of antigen escape. If tumor cells lose one antigen, they may still retain expression of the other, allowing the TCE to bind through the remaining tumor-targeting arm and mediate cytotoxicity. Conversely, when both target antigens are co-expressed, the TCE can engage them simultaneously, resulting in higher-avidity binding and enhanced tumor cell killing (98).

Despite challenges posed by molecular complexity and tumor heterogeneity, TriTCEs are poised to become a cornerstone of solid tumor therapy through intelligent activation strategies and combination therapies.

#### 4.1.2 Conditional activation technique

Conditional activation technology is a key strategy to enhance TCE safety and efficacy by precisely controlling TCE activity in space and time, thereby reducing toxicity.

Spatially controlled activation exploits tumor-specific proteolytic enzymes to enable localized drug activation. For example, the ProTriTAC platform incorporates substrate sequences cleavable by matrix metalloproteinases (MMP-2/9) or urokinase plasminogen activator, which are overexpressed in solid tumors but minimally expressed in healthy tissues (99). This design ensures TCE activation primarily occurs at tumor sites. Preclinical models demonstrated markedly reduced off-target T cell activation in normal organs compared to conventional TCEs. Similarly, Janux’s TRACTr platform employs MMP-responsive masking peptides that block CD3-binding domains until cleaved within the TME, enabling tumor-selective T cell engagement (100). TCEs using this masking peptide technology are currently in clinical trials.

Temporally controlled activation utilizes systemic proteases for gradual prodrug conversion, minimizing CRS risk. TriTAC-XR exemplifies this approach (101). It features an N-terminal peptide mask and a protease-cleavable linker that block CD3 binding until slowly activated by systemic proteases. *In vitro*, the masked prodrug exhibited reduced CD3 affinity and T cell-dependent cytotoxicity compared to its active form. In cynomolgus monkeys, TriTAC-XR significantly lowered cytokine production while maintaining comparable pharmacodynamic effects and improved Cmax/Cmin ratios versus the unmasked drug. Unlike tumor-activated prodrugs, TriTAC-XR harnesses systemic proteases for time-dependent activation, enabling sustained drug release. This extended-release mechanism combines the safety benefits of subcutaneous dosing (reduced CRS risk) with the convenience of intravenous administration (lower immunogenicity). By prolonging drug exposure, TriTAC-XR may also allow less frequent dosing. These features position TriTAC-XR as a promising strategy to expand the therapeutic window of TCEs by balancing efficacy with improved tolerability.

In addition, conditional activation technologies that exploit the unique chemical environment of the TME have made significant advances, exemplified by Xilio’s AnTenGager. This platform employs a pH-sensitive linker designed to specifically recognize the acidic TME (pH 6.5-6.9) (102). Upon reaching the TME, the acidic pH triggers linker cleavage, releasing the masking peptide and thereby activating the antitumor function. Preclinical data demonstrated substantially higher drug activity in tumor tissue versus plasma, indicating enhanced tumor specificity and reduced off-tumor toxicity. Other pH-responsive activation strategies are also under active investigation, including conformational changes (BioAtla’s EpCAMxCD3 bispecific antibody) and affinity gradient modulation (VelicicityBooster’s LBL-034) (103, 104). Several agents employing these approaches have entered clinical evaluation, underscoring their translational potential in solid tumor therapy.

Conditional activation technologies resolve the toxicity-efficacy paradox of conventional TCEs through spatial, temporal, and chemical environment-specific control. Platforms such as ProTriTAC, TRACTr, TriTAC-XR, and AnTenGager exemplify diverse design strategies with promising preclinical validation. Further optimization of these response mechanisms is necessary to enable personalized tumor immunotherapy.

### 4.2 The combination therapy development

While TCEs offer new promise for solid tumor immunotherapy, monotherapy remains limited. Combination therapies may overcome challenges such as drug resistance and insufficient T cell activation.

#### 4.2.1 Combination with ICIs

Studies have shown that the antitumor efficacy of TCEs correlates positively with baseline CD8+ T cell density in the TME; “cold tumors” with low T cell infiltration exhibit resistance to TCEs (105). Furthermore, combining TCEs with ICIs or a 4-1BB agonist significantly enhanced T cell responses, resulting in a 32-fold expansion of CD8+ T cells within the TME and improved efficacy. Key factors contributing to TCE resistance include insufficient baseline T cell infiltration and treatment-induced limitations on T cell recruitment, while preferential CD8+ T cell expansion marks successful treatment response. The combination of TCEs and ICIs enhances antitumor activity via multiple mechanisms, including synergistic T cell activation and remodeling of the TME. TCEs, through their bispecific binding, directly activate and guide T cells to tumor cells (106). Concurrently, ICIs block inhibitory checkpoints such as PD-1, PD-L1, and CTLA-4, relieving immunosuppression, restoring T cell function, and delaying exhaustion (107). This dual approach provides both “strong activation” and “immunosuppression relief”, substantially boosting T cell-mediated tumor killing. Additionally, combination therapy can overcome TME immunosuppressive barriers and mitigate single-agent resistance by targeting multiple pathways.

Recent clinical trials have explored Blinatumomab combined with PD-1 inhibitors. In a Phase I/II trial (NCT03340766) for relapsed/refractory diffuse large B cell lymphoma (DLBCL), Blinatumomab plus Pembrolizumab achieved an ORR of 60% and a CR rate of 35%, outperforming Blinatumomab monotherapy (ORR 30-40%). Median PFS improved to 5.2 months versus 2.6 months with Blinatumomab alone. Patients with PD-L1 expression ≥50% had higher CR rates (50% vs. 20%) (108). In another trial for relapsed/refractory B-ALL (NCT03160079), Blinatumomab plus Nivolumab increased ORR to 56% and minimal residual disease negativity to 89% (versus 76% with Blinatumomab alone), with median OS extended to 11.2 months from 7.7 months. CD19 antigen loss rates decreased from 15% to 5% (109). While TCE monotherapy has proven effective in hematologic malignancies, combination therapy with ICIs is primarily targeted at relapsed or refractory cases.

Two multicenter phase I clinical trials (NCT02324257 and NCT02650713) evaluated the safety and efficacy of the bispecific antibody cibisatamab, targeting CEA and CD3 simultaneously, either as monotherapy or combined with the PD-L1 inhibitor atezolizumab, in CEA-positive solid tumors, predominantly colorectal cancer (CRC) (110–112). In the monotherapy cohort (n=149), doses escalated to 600 mg weekly, while the combination cohort (n=228) received cibisatamab (5-160 mg weekly) alongside atezolizumab (1200 mg every 3 weeks). The ORR was 4% in the monotherapy group and 5% in the microsatellite-stable CRC (MSS-CRC) subgroup. The combination group demonstrated an improved overall ORR of 7%, with a notable 14% ORR in the MSS-CRC subgroup at a fixed dose, surpassing historical ORRs of 2% for monotherapy or atezolizumab alone, indicating synergistic effects. Regarding safety, grade ≥3 TrAEs occurred more frequently in the combination group (49%) than in monotherapy (36%). Common adverse events included fever, diarrhea, infusion reactions, and immune-related toxicities such as colitis, which were generally manageable. The safety profile supports further validation in phase II trials.

In a phase III clinical trial involving 378 patients with HLA-A*02:01-positive mUM receiving first-line treatment, patients were randomized to receive either tebentafusp (tebe, n=252) or investigator’s choice therapy (IC, n=126; including pembrolizumab 82%, ipilimumab 12%, or dacarbazine 6%) (113). Data from the first interim analysis in November 2020 showed that in the ICIs follow-up treatment subgroup, the tebe group achieved a mOS of 16 months (1-year OS rate 63%), significantly better than the IC group’s mOS of 9 months (1-year OS rate 47%) (HR = 0.62, 95% CI 0.34-1.14), and superior to historical second-line treatment benchmarks (mOS 7 months, 1-year OS rate 35%). These findings suggest that patients receiving ICIs after progression on tebe as first-line therapy had significantly improved OS compared to those receiving ICIs following IC therapy. This may be due to tebentafusp’s ability to provide a more sensitive immune target by activating T cells and remodeling the TME. Although confounding factors such as baseline characteristic differences remain, these results provide a rationale for combination therapies in tebentafusp as a first-line treatment for mUM.

Several clinical trials are currently investigating the combination of TCEs with ICIs or 4-1BB agonists in solid tumors, including NCT04740034 and NCT03564340 (114, 115). Furthermore, bispecific TCEs targeting PD-L1 and CD3 have been developed, demonstrating increased T lymphocyte infiltration and inhibition of tumor proliferation without peripheral T-cell activation in melanoma (A375) xenograft models (116, 117).

In conclusion, combining TCEs with ICIs offers a promising immunotherapeutic strategy for solid tumors; however, larger clinical trials and improved toxicity management are needed to advance clinical application.

#### 4.2.2 Combination with oncolytic viruses

Oncolytic viruses selectively infected and lysed tumor cells, releasing TAAs, activating dendritic cells (DCs), and facilitating antigen presentation, as well as inducing secretion of IFN-α/β, which recruited naturally occurring immune cells to the TME (118). Simultaneously, TCE expanded killing of uninfected tumor cells by activating T cells. The combination with oncolytic viruses achieved a synergistic effect of direct tumor cell lysis and immune cell-mediated killing.

Several drugs made breakthroughs in preclinical trials. VG21306 is a novel oncolytic virus genetically engineered to encode a secretable BiTE targeting carcinoembryonic antigen cell adhesion molecule 6 (CEACAM6) on tumor cells (119). This design overcame physical barriers of solid tumors and released BiTE locally within the tumor, avoiding off-target toxicity. In terms of safety, no measurable BiTE leakage was detected in the blood after intratumoral injection in mice. BiTE showed no binding activity to normal tissues, indicating precise local enrichment. The innovation of VG21306 combined oncolytic virus-mediated tumor lysis with immune activation via BiTE, providing a therapeutic strategy with targeting, safety, and broad-spectrum killing potential for CEACAM6-positive solid tumors such as colorectal and pancreatic cancer. It also overcame physical barriers of solid tumors, potentially addressing insufficient penetration and systemic toxicity challenges of traditional immunotherapy. In another study, safety and antitumor activity improved significantly by loading an oncolytic adenovirus expressing BiTE onto mesenchymal stem cells (MSCs) (120). In a hepatocellular carcinoma mouse model, tumor volume was reduced by more than 50% compared to traditional immunotherapy. In a heterogeneous model with mixed AFP-positive and -negative cells, this approach efficiently eliminated AFP-negative tumor cells, overcoming targeting limitations of traditional immunotherapy. Furthermore, due to the precise delivery by MSCs, extrahepatic organ damage did not increase, and liver function indices decreased significantly, suggesting reduced hepatotoxicity.

TCEs combined with oncolytic viruses generated immune memory and induced long-term efficacy (121). Oncolytic virus–mediated tumor lysis released TAAs that memory T cells rapidly expanded upon antigen re-encounter. The virus induced epitope spreading, activating memory cells targeting new epitopes and broadening protection (122). Tumor cells infected by the oncolytic virus released type I interferons such as IFN-α/β, enhancing DC maturation and T cell memory potential (123). An experiment showed that cured mice exhibited complete protection against homologous tumor rechallenge, indicating generation of durable memory T cells (124).

## 5 Immune cell engagers beyond T cells

In addition to TCEs, the field of immune cell engagers has expanded to include bispecific and multispecific antibodies that redirect immune cells beyond T cells, particularly NK cells, macrophages, and other innate immune effectors (125). These platforms have attracted growing interest for solid tumors, which often exhibit poor T cell infiltration, T cell exhaustion, defects in antigen presentation, and treatment-related toxicities. Although non-T-cell engagers are generally less clinically advanced than TCEs, their rapid development underscores the broader potential of immune cell redirection.

Among these emerging modalities, NK cell engagers are the most extensively studied. These agents typically contain one binding domain targeting a tumor-associated antigen and another targeting an activating NK cell receptor, such as CD16a, NKp30, or NKG2D, to promote NK cell–mediated cytotoxicity against tumor cells (126, 127). Compared with TCEs, NK cell engagers may offer several potential advantages in solid tumors. Unlike T cell–based therapies, which often require sufficient infiltration and preserved function of tumor-reactive T cells, NK cell engagers can recruit innate immune effectors capable of rapid cytotoxic responses without prior antigen sensitization (128). This feature may be especially valuable in tumors with limited T cell infiltration or profound T cell exhaustion. In addition, NK cell–mediated killing is less dependent on intact antigen presentation machinery, enabling these agents to remain active in tumors that evade adaptive immunity through loss of MHC expression or defects in antigen processing and presentation (129). Upon activation, NK cells directly lyse tumor cells via perforin/granzyme release and death receptor pathways, while also secreting cytokines such as IFN-γ, which can enhance dendritic cell activation and foster broader antitumor immunity. Furthermore, because NK cell engagers may induce less systemic immune activation than direct T cell redirection, they could potentially reduce the risk of severe CRS. Collectively, these features position NK cell engagers as an attractive strategy for immunologically “cold” tumors and for those that have developed adaptive resistance to T cell–based therapies.

Macrophage engagers and myeloid cell engagers have also emerged as promising strategies for solid tumors (125). Rather than primarily driving direct T cell–mediated killing, these approaches harness innate immune functions such as phagocytosis, cytokine production, and remodeling of the TME. By redirecting or reprogramming macrophages and related myeloid cells, they can enhance tumor clearance, promote antigen release, and support broader immune activation, features that may be especially beneficial in solid tumors marked by stromal exclusion, poor T cell infiltration, and immunosuppression.

Importantly, non-T-cell engagers should be viewed not only as alternatives to TCEs but also as potential partners in combination regimens. Different immune effector cells vary in trafficking patterns, activation thresholds, persistence, susceptibility to exhaustion, and toxicity profiles; leveraging these differences may help overcome key barriers to TCE efficacy in solid tumors while simultaneously reshaping the TME.

Nevertheless, significant challenges remain. The abundance, phenotype, and functional state of NK and myeloid cells differ across patients and tumor types. Moreover, excessive activation of innate immunity may still trigger systemic inflammation or off-tumor effects. Future progress will depend on improved target selection, optimized molecular formats, conditional activation strategies, and rational combinations with ICIs, cytokine modulation, or other TME-directed therapies.

## 6 Biomarkers for patient stratification and response prediction

A major challenge in the clinical development of TCEs for solid tumors is identifying biomarkers that can refine patient selection, predict therapeutic benefit, and enable early monitoring of efficacy and safety. Because TCE activity depends on the presence of an actionable tumor target, a permissive immune microenvironment, and sufficient host T cell competence, biomarker strategies should integrate tumor-related, immune-related, and pharmacodynamic features rather than rely on a single parameter.

### 6.1 Tumor-related biomarkers

Tumor-related biomarkers primarily assess whether tumor cells express the molecular prerequisites for recognition by TCEs. Among these, target antigen expression level represents the most fundamental selection criterion (74). TCEs can efficiently redirect T cells only when the target antigen is present at sufficient density on tumor cells; thus, both the intensity and uniformity of antigen expression are likely to influence clinical activity. Similarly, for TCEs that recognize intracellular antigents via peptide–HLA complexes, such as PRAME-directed platforms, eligibility also requires the appropriate HLA genotype to support effective antigen presentation.

Beyond mere target positivity, the intrinsic immune phenotype of the tumor may further refine patient selection. Emerging evidence suggests that tumors exhibiting both high target antigen expression and abundant CD8^+^ T cell infiltration, commonly termed “hot tumors”, are most likely to benefit from TCE therapy. In contrast, tumors with adequate target expression but limited endogenous T cell infiltration may respond poorly due to insufficient effector T cell availability within the tumor.

### 6.2 TME and host immune status

The efficacy of TCEs is highly dependent on the functional capacity of the host immune system, particularly the availability and quality of redirectable T cells (64). Within the TME, CD8^+^ T cell density serves as a key predictive parameter, reflecting the abundance of local effector cells available for cytotoxic recruitment. In addition, the CD8^+^/Treg ratio may offer further insight into the local immune balance; a higher ratio generally indicates a less suppressive microenvironment and is often associated with improved clinical response.

Beyond cell numbers, recent studies have emphasized the importance of T cell fitness–related features. Notably, a T cell adaptive signature linked to clinical benefit has been identified, characterized by elevated expression of costimulatory molecules such as CD28, chemokine receptors involved in tumor homing (e.g., CXCR3) (130), and transcriptional programs associated with self-renewal and sustained effector function. These findings suggest that the qualitative state of the T cell compartment may be as critical as its abundance in determining responsiveness to TCEs.

### 6.3 Pharmacodynamic and activity-related biomarkers

Pharmacodynamic biomarkers are essential for assessing whether a TCE exerts its intended biological effects and for monitoring treatment-related toxicities. Among these, cytokine release represents one of the most clinically relevant parameters. Elevated levels of inflammatory cytokines such as IL-6 and IFN-γ indicate immune activation but may also signal the onset of CRS (131). Consequently, cytokine profiles can serve both as markers of on-treatment activity and as early warning signs of toxicity risk.

Effective therapy may also lead to immune cell activation and remodeling within the TME. For instance, successful TCE treatment can induce expansion or phenotypic shifts in antitumor immune populations, including dendritic cells and M1-like macrophages, alongside enhanced activation of effector T cells.

### 6.4 Exploratory and composite biomarkers

Because the predictive value of any single biomarker is often limited, increasing attention has focused on composite biomarker models. Quantitative systems pharmacology (QSP) and other integrative approaches can combine multiple variables, such as target antigen density, T cell infiltration, CD8^+^/Treg ratio, and pharmacodynamic immune changes, to improve prediction of clinical response (132). Such multidimensional models may be particularly valuable in solid tumors, where therapeutic outcomes depend on the interplay among tumor biology, immune context, and drug-specific factors.

In addition, blood-based monitoring offers a promising complementary strategy, especially when repeated tumor biopsies are impractical. Serial blood sampling enables dynamic assessment of circulating immune cell subsets, their activation states, and treatment-induced immune remodeling. For example, the frequency of circulating naïve CD4^+^ T cells has been proposed as a potential predictive biomarker. Although these blood-based markers remain exploratory, they provide an attractive, minimally invasive option for longitudinal monitoring during TCE therapy.

Taken together, biomarker development for TCE therapy in solid tumors should extend beyond simple target positivity to encompass a broader framework that includes tumor antigen status, tumor immune phenotype, host T cell competence, and dynamic pharmacodynamic changes. Such an integrated strategy could enhance patient selection, support early response assessment, and guide the rational optimization of TCE therapy.

## 7 Conclusion and prospect

TCEs have emerged as a highly promising class of immunotherapy. Although their clinical success was first established in hematologic malignancies, recent advances in molecular engineering and encouraging clinical signals demonstrate that TCEs can also achieve meaningful activity in solid tumors. The recent approval of tarlatamab for the treatment of SCLC marked a key milestone, underscoring the potential of TCEs in this setting.

Nevertheless, applying TCEs to solid tumors remains considerably more challenging than in hematologic malignancies. Heterogeneous antigen expression, antigen loss, poor tumor penetration, an immunosuppressive TME, impaired antigen presentation, T cell dysfunction, and treatment-related toxicities collectively limit the depth, durability, and breadth of response. These barriers highlight that successful TCE therapy in solid tumors depends not only on target recognition but also on the careful integration of tumor biology, immune context, and molecular design.

Rapid progress in next-generation formats offers multiple opportunities to address these limitations. Improved architectures, including 2 + 1 avidity-based designs, trispecific molecules, and conditional or tumor-activated constructs, are expanding both the therapeutic window and the range of viable targets. Concurrently, deeper insights into resistance mechanisms emphasize the importance of biomarker-guided development, encompassing assessment of antigen density and homogeneity, MHC-I integrity, T cell fitness, cytokine dynamics, and features of the TME. Rational combinations with checkpoint inhibitors, cytokine modulation, stromal-targeting approaches, and other microenvironment-directed therapies may further enhance efficacy and durability.

Beyond continued optimization of TCEs, emerging engager platforms that redirect NK cells, macrophages, and other innate immune cells could broaden the therapeutic landscape of solid tumor immunotherapy.

Overall, the future of TCE therapy in solid tumors will likely hinge on more precisely engineered, biomarker-informed, and context-specific strategies. With ongoing advances in target selection, molecular design, toxicity management, and combination therapy, TCEs and related engager platforms are poised to become an increasingly integral component of the therapeutic arsenal against solid tumors.
